# A Complex Case of Acalculous Cholecystitis, Cholangitis, and Pancreatitis Managed With Percutaneous Cholecystostomy in a Septic Patient

**DOI:** 10.7759/cureus.89636

**Published:** 2025-08-08

**Authors:** Sahil Patel, Jeffrey Joppen, Syed Rahman, Divya Sheth, Achal Patel

**Affiliations:** 1 Internal Medicine, Nova Southeastern University Dr. Kiran C. Patel College of Osteopathic Medicine, Fort Lauderdale, USA; 2 Gastroenterology and Hepatology, Nassau University Medical Center, East Meadow, USA

**Keywords:** acute acalculous cholecystitis (aac), acute cholangitis, acute pancreatitis, biliary diseases, percutaneous cholecystostomy tube, severe sepsis

## Abstract

This case report presents a complex case of acute cholecystitis, cholangitis, pancreatitis, intrahepatic abscesses, and sepsis without biliary obstruction, highlighting the challenges of managing multi-organ involvement in a critically ill individual. The patient, a middle-aged male, presented with fever, jaundice, and abdominal pain, with imaging revealing biliary ductal dilation, a distended gallbladder, and a staghorn calculus. Laboratory findings showed elevated liver enzymes, bilirubin, and lipase, supporting the diagnosis of acute cholecystitis, cholangitis, and pancreatitis. Given the severity of his condition, characterized by septic shock and acute kidney injury, percutaneous cholecystostomy (PCT) was chosen over surgical intervention to provide biliary drainage and infection control while avoiding the morbidity associated with invasive procedures. The patient’s clinical course improved with PCT, targeted antibiotics, and supportive care. This case underscores the complexity of managing multi-organ dysfunction and highlights the potential of PCT as a viable alternative to surgery in critically ill patients with severe cholecystitis and cholangitis with intrahepatic abscesses. The simultaneous occurrence of these conditions in the context of no biliary obstruction is uncommon, and this case offers insight into treatment strategies for such complex presentations. Future research should compare the outcomes and symptomatology of PCT versus surgery in similar critically ill populations to refine management approaches and optimize patient outcomes.

## Introduction

Percutaneous cholecystostomy (PC) is a minimally invasive procedure that involves the percutaneous insertion of a drainage catheter into the gallbladder to relieve biliary obstruction and inflammation [1]. It is primarily indicated in patients with acute cholecystitis who are poor surgical candidates due to advanced age, significant comorbidities, or hemodynamic instability [2]. In these patients, PC serves as an effective alternative to cholecystectomy by providing temporary biliary decompression and infection control, either as a bridge to surgery or as definitive management.

The procedure is typically performed under image guidance, utilizing ultrasound, computed tomography (CT), or fluoroscopy. Ultrasound-guided PC is often preferred due to its real-time visualization, absence of ionizing radiation, and ability to assess surrounding structures dynamically [3]. However, in cases where ultrasound imaging is limited due to bowel gas or patient body habitus, CT or fluoroscopic guidance may be employed to ensure precise catheter placement. While PC is widely used for the management of acute calculous and acalculous cholecystitis, its role in complex hepatobiliary conditions involving concurrent cholangitis, pancreatitis, and sepsis is less well-documented. The presence of a staghorn calculus, an extensive branching stone that fills the renal pelvis and calyces, adds another layer of complexity, as it can lead to significant obstruction, recurrent infections, and progressive inflammation involving multiple organ systems. In such cases, the interaction between biliary obstruction and pancreatic inflammation creates a challenging clinical scenario, necessitating a multidisciplinary approach to management [1,4].

This case report highlights the clinical complexity of a patient presenting with concurrent acalculous cholecystitis, cholangitis, and pancreatitis in the setting of sepsis and discusses the role of PC in stabilizing critically ill patients. While each condition is commonly encountered, their simultaneous presentation posed diagnostic and management challenges. The case underscores the importance of individualized, multidisciplinary care in guiding timely, minimally invasive intervention.

## Case presentation

Patient history and physical examination

A 62-year-old male with no significant medical history presented to the emergency department with severe abdominal pain. The pain had started suddenly the previous afternoon after consuming soup and was described by the patient as sharp and persistent, primarily localized to the epigastric and right upper quadrant (RUQ) regions. The patient also reported associated symptoms, including nausea, vomiting, chills, and subjective fever. He denied any history of gallstones, biliary disease, nonsteroidal anti-inflammatory drug use, alcohol abuse, gastrointestinal bleeding, or pancreatitis. Additionally, there were no reports of recent travel, antibiotic use, sick contacts, or significant weight loss. His diet and lifestyle had not changed recently, and he denied other gastrointestinal or constitutional symptoms. On presentation to the emergency department, the patient was alert and oriented but appeared uncomfortable. His vital signs were as follows: blood pressure 130/70 to 150/90 mmHg, heart rate between 50 and 70 beats per minute, respiratory rate of 16 breaths per minute, temperature 98.5°F, and oxygen saturation of 98% on room air. The physical examination revealed mild abdominal distension, with significant tenderness to palpation in both the epigastric and RUQ regions. A positive Murphy’s sign was noted, which suggested the possibility of cholecystitis. There was no peritoneal irritation, as no rebound tenderness or guarding was observed. The patient’s cardiovascular and respiratory exams were unremarkable. There were signs of jaundice, fluid retention, or peripheral edema.

Laboratory findings

Laboratory studies were ordered to assess the patient’s condition. The complete blood count showed a markedly elevated white blood cell count of 32 × 10⁹/L, indicating a significant inflammatory response. Hemoglobin was found to be 11.1 g/dL, decreased from his baseline of 14.2 g/dL, which raised concern for possible blood loss, dehydration, or hemoconcentration. Platelet count was reduced to 79 × 10⁹/L, down from 267 × 10⁹/L, suggesting thrombocytopenia likely related to septicemia. Liver function tests revealed significantly elevated alanine aminotransferase at 343 U/L, aspartate aminotransferase at 428 U/L, and alkaline phosphatase, along with a mildly elevated total bilirubin level. These results were suggestive of hepatic involvement, likely due to cholangitis or biliary obstruction. The lipase level was markedly elevated at >2,650 U/L, confirming the presence of acute pancreatitis. Renal function was also affected, with an elevated blood urea nitrogen level of 39 mg/dL and serum creatinine of 2.6 mg/dL, indicating acute kidney injury. Coagulation studies showed an increase in the international normalized ratio from 1 to 1.7, which suggested a mild coagulopathy likely related to liver dysfunction or sepsis (Table 1).

**Table 1:** Lab results ordered during hospital admission. All undocumented lab findings were in the normal range. WBC: white blood cell; ALT: alanine aminotransferase; AST: aspartate aminotransferase; BUN: blood urea nitrogen; INR: international normalized ratio.

Lab Test	Results	Reference Range
WBC	32 × 10⁹/L	40-110 × 10⁸/L
Hemoglobin	11.1 g/dL	13.5-17.5 g/dL
Platelets	79 × 10⁹/L	150 × 10⁹-450 × 10⁹/L
ALT	343 U/L	7-56 U/L
AST	428 U/L	10-40 U/L
Lipase	2650 U/L	0-160 U/L
BUN	39 mg/dL	7-20 mg/dL
Creatinine	2.6 mg/dL	0.74-1.35 mg/dL
INR	1.7	0.8-1.2

Imaging studies

**Figure 1:**
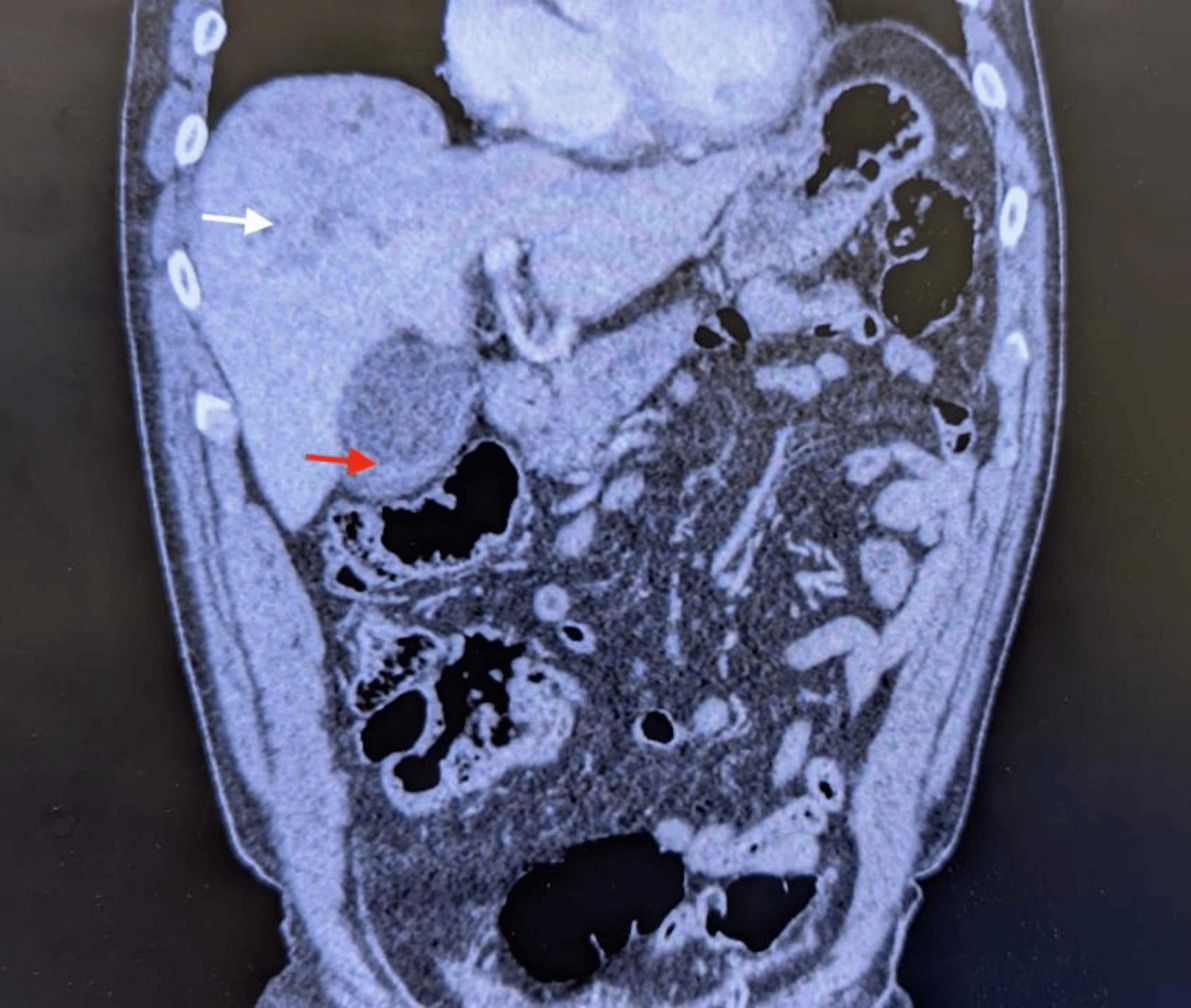
Patient's abdominal CT showing intrahepatic lesions (white arrow) and cholecystitis (red arrow).

**Figure 2:**
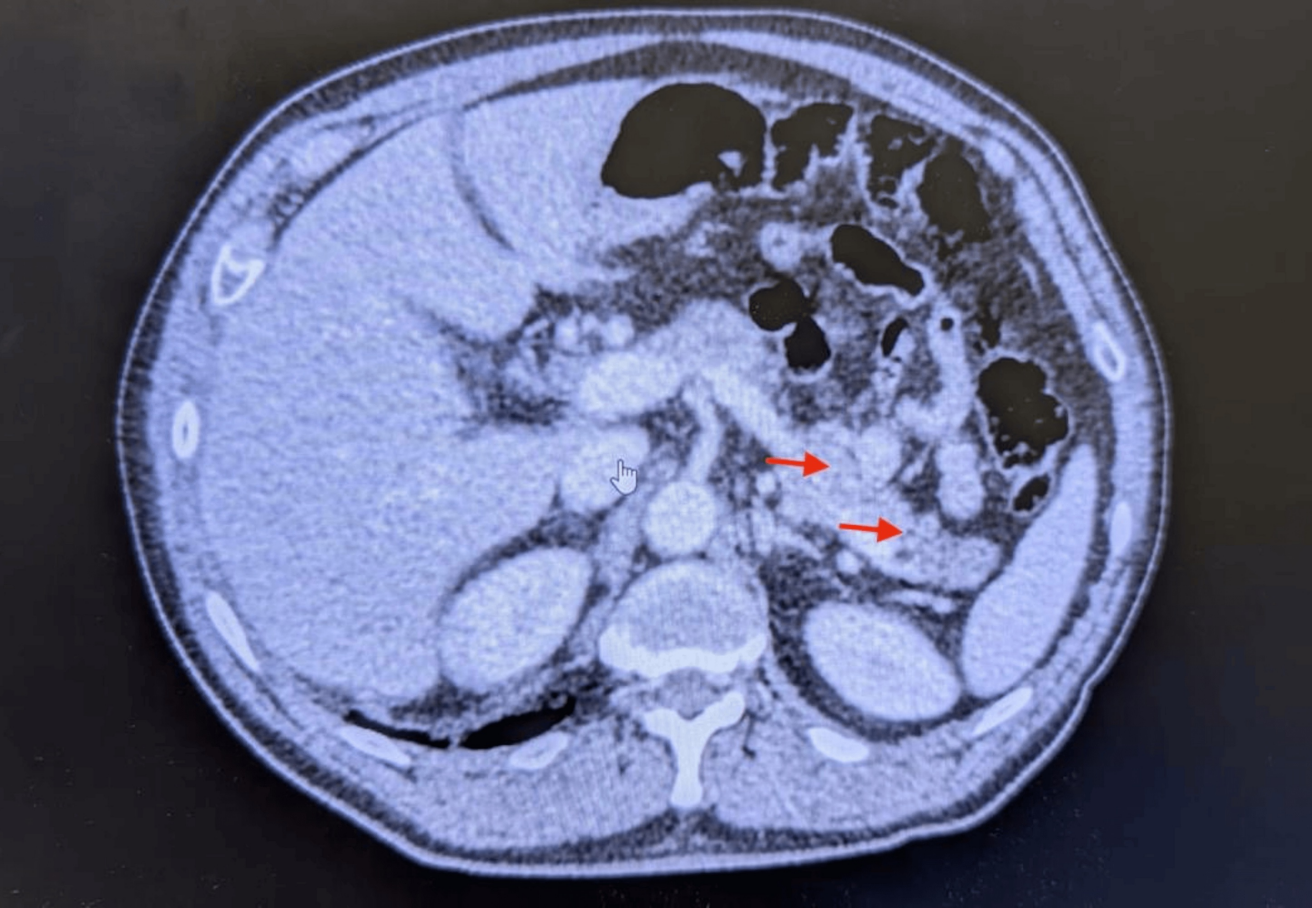
Patient's abdominal CT showing pancreatitis (red arrows).

Several imaging studies were performed to further investigate the patient's symptoms. An abdominal ultrasound was conducted, which revealed gallbladder wall thickening, intrahepatic biliary dilation, and mild hepatomegaly, but no gallstones were identified. This raised suspicion of acute acalculous cholecystitis (AAC). Given the complexity of the clinical presentation, a magnetic resonance cholangiopancreatography (MRCP) was ordered. The MRCP findings revealed pararenal fat stranding, indicative of pancreatitis, along with multiple hepatic lesions suggestive of cholangitis. Additionally, the MRCP showed a dilated common bile duct, which pointed toward biliary obstruction. An abdominal CT scan with contrast was also performed to assess the full extent of the patient’s condition. The CT scan demonstrated cholecystitis, pancreatitis, and the presence of a staghorn calculus in the right kidney with associated moderate hydronephrosis. Multiple hypodense hepatic lesions were observed (Figures 1, 2). This imaging confirmed the presence of multiple organ involvement, including complicated cholecystitis, cholangitis, pancreatitis, and the presence of a staghorn calculus, all contributing to the patient's deteriorating clinical condition (Table 2).

**Table 2:** Imaging studies and findings from the patient case.

Image Modality	Clinical Findings
Abdominal ultrasound	Gallbladder wall thickening, intrahepatic biliary dilation, mild hepatomegaly, but no gallstones were identified
Magnetic resonance cholangiopancreatography	Pararenal fat stranding, multiple hepatic lesions, dilated common bile duct
Abdominal CT scan	Presence of cholecystitis, pancreatitis, a staghorn calculus in the right kidney, moderate hydronephrosis, multiple hypodense hepatic lesions, bilateral pleural effusions

Clinical course

Upon evaluation, the patient was diagnosed with a combination of AAC, severe acute pancreatitis, ascending cholangitis, sepsis, and acute kidney injury (AKI) secondary to obstruction. The decision was made to initiate aggressive intravenous fluid resuscitation and broad-spectrum antibiotics to cover potential pathogens, including both Gram-negative and Gram-positive organisms. Blood cultures and bile cultures were obtained to guide future antibiotic therapy. Once *Escherichia coli* was identified from both blood and bile cultures, the antibiotic regimen was narrowed to specifically target this organism. Due to the patient's critical condition, a surgical consult was requested, but given the severity of the illness, an elective cholecystectomy was deferred. Instead, an interventional radiology consult was made to explore the possibility of performing a percutaneous cholecystostomy. This approach was chosen over surgery because the patient’s sepsis and hemodynamic instability made surgical intervention too risky at that time.

Interventions

**Figure 3:**
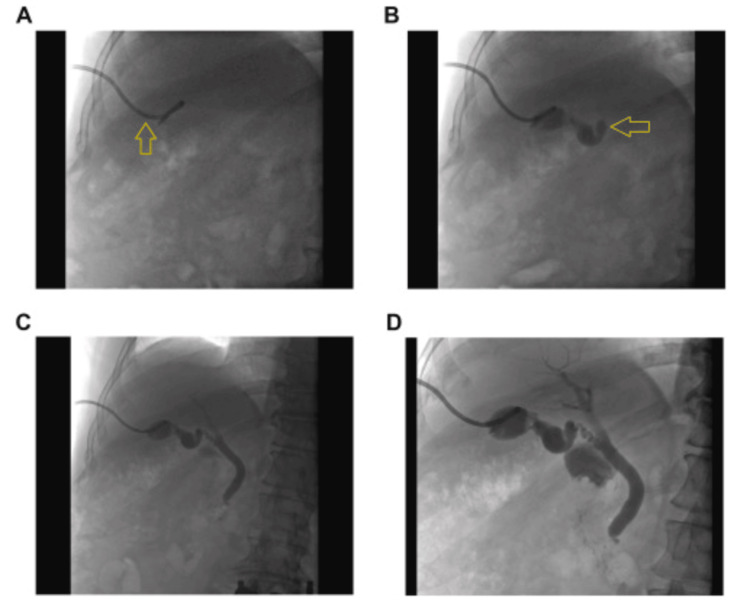
Fluoroscopic image of a cholecystogram after insertion of percutaneous cholecystostomy tube. Reproduced from Kesim and Özen (2023) [5]; licensed under the Creative Commons Attribution 4.0 International License (CC BY 4.0). A, Initial fluoroscopic image showing contrast injection into a percutaneous catheter with limited biliary tree opacification, and initial placement of the percutaneous cholecystostomy tube within the gallbladder (yellow arrow). B, Contrast begins to fill the intrahepatic ducts, outlining segmental biliary structures with evidence of distal obstruction, and drainage of purulent bile through the tube (yellow arrow), confirming successful decompression. C, Further opacification of the biliary system demonstrates partial visualization of the common bile duct and improved proximal flow. D, Complete opacification of the intrahepatic and extrahepatic bile ducts confirms successful biliary drainage and catheter positioning.

A percutaneous cholecystostomy was performed by interventional radiology. A pigtail catheter was inserted into the gallbladder to allow drainage of bile and relieve gallbladder distention. This minimally invasive procedure was chosen to stabilize the patient’s condition before any further surgical intervention could be considered. Following the procedure, the patient was transferred to the intensive care unit (ICU) for close monitoring, given the severity of sepsis and multiple organ involvement. Throughout his ICU stay, the patient was monitored for signs of worsening organ dysfunction, particularly renal failure and coagulopathy. He was kept on a broad-spectrum antibiotic regimen until blood and bile cultures confirmed the presence of *E. coli*, at which point antibiotics were adjusted to target this pathogen specifically. Figures 3A-3D depict a representative fluoroscopic image of a cholecystogram after insertion of percutaneous cholecystostomy tube [5].

Patient outcome and follow-up

The patient gradually improved over the following 48 hours. His fever subsided, and he became afebrile. His vital signs stabilized, with more consistent blood pressure and heart rate. Laboratory results indicated a decline in white blood cell count, which was a positive response to the antibiotics. Renal function also improved, with a decrease in blood urea nitrogen (BUN) and creatinine levels, indicating that the acute kidney injury was resolving. His platelet count normalized, reflecting a resolution of the septic process. By hospital day 10, the patient’s kidney function had returned to baseline, and his creatinine level had normalized. Follow-up imaging studies revealed a reduction in the size of the hepatic abscesses and a decrease in biliary dilation, confirming clinical improvement. The percutaneous cholecystostomy catheter remained in place for two weeks to allow for continued drainage and monitoring. The patient was discharged with instructions for follow-up care. He was advised to return for an elective cholecystectomy once his condition was further stabilized. In the meantime, he continued antibiotic therapy based on culture sensitivities, which was planned to continue until the infection was completely resolved. The patient was also scheduled for a follow-up with nephrology to monitor kidney function and ensure full recovery from the acute kidney injury. Furthermore, he was instructed to undergo regular outpatient care to monitor his liver function, and a repeat ultrasound was planned to assess ongoing biliary and hepatic status.

## Discussion

This case illustrates the complex presentation of concurrent acute cholecystitis, cholangitis, pancreatitis, intrahepatic abscesses, and sepsis, which significantly complicated both diagnosis and management. The presentation of multi-organ involvement in this case required a comprehensive and nuanced approach to management. The staghorn calculus, a large, branching stone typically associated with the renal system, was found incidentally in the patient's kidney. While primarily linked to obstructive uropathy and recurrent urinary tract infections, its presence may have contributed to systemic inflammation or served as a nidus for infection, exacerbating the patient’s septic state [6]. Although direct involvement of biliary and pancreatic structures by renal calculi is rare, the co-occurrence of these conditions in this patient raises the possibility of a shared infectious or inflammatory pathway. The underlying sepsis from ascending cholangitis and bacteremia further complicated management, rendering surgical intervention high risk due to hemodynamic instability [7].

The patient's presentation highlighted the importance of early recognition and intervention in critically ill patients with multi-organ dysfunction. The management of sepsis complicated by biliary and pancreatic pathology requires a multidisciplinary team approach, including specialists in hepatobiliary surgery, gastroenterology, radiology, and critical care [7]. This case illustrates how coordinated care and minimally invasive techniques can be leveraged in the face of diagnostic and therapeutic challenges. Prompt imaging, including MRCP and CT, was critical in delineating the extent of the biliary and pancreatic involvement, as well as in guiding the decision for PCT [8]. The imaging findings of biliary ductal dilation, gas locules suggestive of ascending cholangitis, and a distended gallbladder with wall thickening were all consistent with acute cholecystitis and cholangitis [8]. Biliary dilatation is most commonly caused by obstructive pathologies such as choledocholithiasis, biliary strictures, or malignancies affecting the biliary tract. Infectious causes, such as ascending cholangitis, can also result in biliary dilation due to inflammation and impaired bile flow. In this case, although no obstructing biliary stone was identified, the patient’s imaging showed biliary ductal dilation and intrahepatic abscesses, suggesting that infection and inflammation were the primary drivers. The absence of mechanical obstruction highlights the atypical nature of this presentation. However, the elevated lipase levels and findings on imaging were also concerning for pancreatitis, necessitating a careful balancing of treatment approaches to address both pancreatitis and biliary obstruction [8]. While surgery remains the definitive treatment for cholecystitis and cholangitis, this case exemplifies the complexity of deciding when surgery should be deferred in critically ill patients. The use of PCT as an alternative to surgery in such patients has been increasingly reported in the literature. PCT offers a minimally invasive approach to biliary drainage and infection control, reducing the risks associated with major surgical procedures in patients with severe comorbidities [2,9]. The decision to opt for PCT in this patient was based on several factors: the patient's unstable clinical status, the presence of sepsis and organ failure, and the potential complications of major surgery. PCT allowed for the management of the infection and biliary drainage without the immediate need for more invasive procedures, such as cholecystectomy or biliary tract exploration [9]. This case also underscores the importance of careful and continuous monitoring of patients with such complex, multi-system involvement. The patient’s ongoing leukocytosis, fluctuating liver function tests, and deteriorating kidney function leading to AKI highlight the persistent infection and need for aggressive supportive care [8,9]. The success of PCT in this patient, along with targeted antibiotics based on cultures growing *E. coli*, was a critical turning point in his management. This supports the growing body of evidence suggesting that PCT can be an effective intervention for managing acute cholecystitis and cholangitis, especially in high-risk, critically ill patients who may not be candidates for immediate surgery.

Percutaneous cholecystostomy is a well-established intervention in patients who are not surgical candidates, particularly those presenting with sepsis or multi-organ dysfunction. Prospective studies evaluating the outcomes of PCT in patients with severe pancreatitis, acute cholecystitis, and cholangitis, particularly in septic shock, would provide valuable data to refine treatment strategies [10]. While prior literature supports its use, this case adds to existing evidence by reinforcing the importance of individualized decision-making based on hemodynamic stability, imaging findings, and infection control needs. Rather than suggesting a need for broad new guidelines, this case emphasizes the role of multidisciplinary collaboration in selecting appropriate, patient-centered treatment strategies.

## Conclusions

This case of acute acalculous cholecystitis, severe acute pancreatitis, intrahepatic abscesses, and cholangitis, complicated by sepsis and bacteremia, underscores the complexity of managing multi-organ dysfunction in critically ill patients. The decision to choose PCT over surgery was based on the patient’s unstable clinical condition, with the goal of providing biliary drainage and infection control while minimizing the risks of invasive procedures. This decision highlights the importance of individualized treatment strategies that prioritize patient safety, particularly in patients with severe comorbidities who are not candidates for immediate surgery. Moreover, it supports the use of PCT as a viable, minimally invasive alternative to surgery in critically ill patients, particularly when surgical risks are high. Given the growing evidence in support of PCT, future research should focus on comparing the outcomes of PCT versus surgery in critically ill patients with severe cholecystitis, cholangitis, and pancreatitis, especially in the context of septic shock. These studies will help refine treatment protocols and identify the most appropriate interventions based on patient characteristics, clinical severity, and response to initial therapies.
